# *Plasmodium knowlesi* as a model system for characterising *Plasmodium vivax* drug resistance candidate genes

**DOI:** 10.1371/journal.pntd.0007470

**Published:** 2019-06-03

**Authors:** Lisa H. Verzier, Rachael Coyle, Shivani Singh, Theo Sanderson, Julian C. Rayner

**Affiliations:** Parasites and Microbes Programme, Wellcome Sanger Institute, Wellcome Genome Campus, Hinxton, Cambridgeshire, United Kingdom; Fundaçao Oswaldo Cruz, BRAZIL

## Abstract

*Plasmodium vivax* causes the majority of malaria outside Africa, but is poorly understood at a cellular level partly due to technical difficulties in maintaining it in *in vitro* culture conditions. In the past decades, drug resistant *P. vivax* parasites have emerged, mainly in Southeast Asia, but while some molecular markers of resistance have been identified, none have so far been confirmed experimentally, which limits interpretation of the markers, and hence our ability to monitor and control the spread of resistance. Some of these potential markers have been identified through *P. vivax* genome-wide population genetic analyses, which highlighted genes under recent evolutionary selection in Southeast Asia, where chloroquine resistance is most prevalent. These genes could be involved in drug resistance, but no experimental proof currently exists to support this hypothesis. In this study, we used *Plasmodium knowlesi*, the most closely related species to *P. vivax* that can be cultured in human erythrocytes, as a model system to express *P. vivax* genes and test for their role in drug resistance. We adopted a strategy of episomal expression, and were able to express fourteen *P. vivax* genes, including two allelic variants of several hypothetical resistance genes. Their expression level and localisation were assessed, confirming cellular locations conjectured from orthologous species, and suggesting locations for several previously unlocalised proteins, including an apical location for PVX_101445. These findings establish *P. knowlesi* as a suitable model for *P. vivax* protein expression. We performed chloroquine and mefloquine drug assays, finding no significant differences in drug sensitivity: these results could be due to technical issues, or could indicate that these genes are not actually involved in drug resistance, despite being under positive selection pressure in Southeast Asia. These data confirm that *in vitro* *P. knowlesi* is a useful tool for studying *P. vivax* biology. Its close evolutionary relationship to *P. vivax*, high transfection efficiency, and the availability of markers for colocalisation, all make it a powerful model system. Our study is the first of its kind using *P. knowlesi* to study unknown *P. vivax* proteins and investigate drug resistance mechanisms.

## Author summary

*Plasmodium vivax* is the most prevalent human malaria pathogen worldwide, but little is known about its biology as the majority of experimental studies have focused on *Plasmodium falciparum*, the main cause of malaria in Africa. We therefore know little about the underlying mechanisms of drug resistance in *P. vivax* and have yet to develop tools to experimentally understand drug resistance, which limits the ability to adapt drug policies and reduce the spread of resistance. Here, we used a related parasite, *Plasmodium knowlesi*, as a model system to investigate genes that might be involved in *P. vivax* drug resistance. Multiple *P. vivax* proteins were expressed and their location in *P. knowlesi* was explored using organellar markers. None of the candidates induced changes in parasite response to chloroquine or mefloquine, two front-line antimalarials for which resistance is a concern in *P. vivax*. This could be due to technical reasons, or could indicate that these genes do not actually play a role in resistance. Despite not linking candidate gene expression to drug resistance phenotypes, we demonstrated that *P. knowlesi* is a promising model for *P. vivax* studies with rapid generation of transgenic lines and good expression of exogenous *P. vivax* proteins.

## Introduction

*Plasmodium vivax* is the most prevalent cause of human malaria worldwide, with nearly half of humankind living in areas at risk [1–4]. As *P. vivax* only invades reticulocytes [5], which comprise only a small fraction of circulating red blood cells, infections are usually characterised by lower parasitaemia and less severe symptoms than *P. falciparum* infections. Nonetheless, *P. vivax* causes serious morbidity and sometimes mortality [6, 7] and is particularly challenging to eradicate, with *P. vivax* often persisting in areas where *P. falciparum* has been effectively controlled [1, 2, 4]. This is in part because *P. vivax* gametocytes develop rapidly, meaning that transmission can happen before symptoms appear and therefore before treatment is sought. In addition, unlike *P. falciparum*, *P. vivax* has a dormant phase in its life-cycle, the hypnozoite, which can stay inactive in the liver for weeks or months before reactivating and triggering a new infection in the absence of ongoing transmission [8].

Primaquine and tafenoquine are the only drugs active against hypnozoites and both are contraindicated in people with glucose-6-phosphate-dehydrogenase (G6PD) deficiency [9, 10], a common condition in malaria endemic areas, leading to a lack of prescription [9, 11]. More options are available for treatment of blood-stage forms, including chloroquine and mefloquine. Chloroquine, which is no longer used for *P. falciparum* treatment because of widespread resistance, used to be the WHO-approved treatment for uncomplicated *P. vivax* malaria. This changed in 2015 when the first-line treatment in several countries in Southeast Asia was replaced with artemisinin-combination therapy (ACT), due to concerns about high-grade chloroquine resistance. Since the first confirmed case of chloroquine clinical failure in *P. vivax* in 1989 in Papua New Guinea [12], resistance to chloroquine has been detected in a number of locations across Southeast Asia and Latin America [13–21]. Despite the spread of resistance, chloroquine remains the first-line antimalarial in Latin America and the majority of Asia [22]. In addition, *P. vivax* antifolate resistance has been described for more than 50 years [23–27], and more recently mefloquine resistance has also been described in South America and Southeast Asia [18, 28, 29] even though these drugs were never recommended as first-line treatment for *P. vivax* [22].

Despite growing interest in *P. vivax* drug resistance, the underlying cellular mechanisms remain poorly understood, largely due to the absence of a well-established and reproducible *in vitro* culture system for *P. vivax*. Many hypotheses about *P. vivax* resistance mechanisms have therefore come from transposition of knowledge from *P. falciparum*. Such transposition seems informative for antifolate resistance, with SNPs in *pvdhfr* and *pvdhps* associated with decrease in the susceptibility of *P. vivax* to sulfadoxine and pyrimethamine [30–32], just as the homologous genes are involved in antifolate resistance in *P. falciparum*. Similar commonalities are observed for mefloquine, with amplification of *pvmdr1*, the homologue of the major gene associated with *P. falciparum* mefloquine resistance, found in areas where mefloquine was the first-line treatment for *P. falciparum* [33]. The mechanism of chloroquine resistance, however, may be different between the two *Plasmodium* spp. as chloroquine resistance reversal agents for *P. falciparum* seem to be ineffective against *P. vivax* [34]. Several studies have focused on *pvmdr1* and *pvcrt* as possible *P. vivax* chloroquine resistance candidates, based on the role that homologues of these genes play in *P. falciparum*, but the findings have been contradictory. While several field studies did not find any correlation between *pvmdr1* mutation/amplification and chloroquine sensitivity [20, 21, 35–37], Suwanarusk et al. found SNPs in *pvmdr1* correlated with reduced chloroquine sensitivity, and also observed that higher copy numbers of this gene correlated with higher sensitivity [19, 38]. By contrast, Melo et al. observed higher transcription rate of *pvmdr1* in clinically resistant *P. vivax* compared to sensitive isolates [39]. Similar contradictory observations have been made for *pvcrt* with some studies ruling out the gene’s involvement in chloroquine resistance [19–21, 36], while a study from Silva et al. reported higher *pvcrt* copy number in 33% of chloroquine resistant isolates [40]. These discrepancies highlight a lack of understanding of the mode of action of chloroquine on *P. vivax* and the need to develop new *P. vivax* specific techniques for experimentally testing potential molecular markers of resistance.

An alternative approach to identify genes associated with *P. vivax* drug resistance is to identify genes under recent positive selection pressure in parts of the world where drug resistance is known to occur. While many factors could lead to positive selection on a given gene, searching for signatures of positive selection across the *P. falciparum* genome repeatedly identifies known drug resistance genes such as *pfcrt*, *pfmdr1*, *pfdhfr* and *pfdhps* as among the most strongly positively selected genes [41]. A recent study of 200 *P. vivax* whole genome sequences, primarily from Southeast Asia, identified multiple genes under positive selection pressure [42]. These are enticing candidates for drug resistance markers, but clearly require experimental validation before they can be used to provide actionable information about the spread and extent of drug resistance. The lack of a *P. vivax* continuous *in vitro* culture system is a major impediment to such validation. *Ex vivo* schizont maturation assays are the most reliable drug susceptibility assays used for *P. vivax* and allow drug resistance monitoring [43]. In theory these could be used to link resistance phenotype to genotype, but this approach is challenging because *ex vivo* assays are performed on clinical isolates, where genetic diversity is higher. Clinical isolates will differ across tens of thousands of nucleotides, including locations that are not related to drug resistance but could be linked to transmission or simply represent stochastic variation.

Although *in vivo* models using *Aotus* monkeys as a host for *P. vivax* parasites have been a valuable tool for studying the parasite [44, 45], they require costly facilities and significant ethical considerations which limits their use to only a few laboratories, and suffer some of the same drawbacks as *ex vivo* assays because they rely on clinical strains adapted for propagation in monkeys. An alternative approach to bypass the problem of field variability would be to use well-characterised lab strains whose genome can be engineered. Genetic modifications are therefore controlled in order to precisely identify which genotype is mechanistically responsible for the drug resistance phenotype. This is not currently possible for *P. vivax* due to the lack of *in vitro* culture systems, so other *Plasmodium* species must be used as an *in vitro* model. *P. falciparum* *in vitro* culture is carried out by many labs around the world, but its utility as a model for *P. vivax* is limited by the phylogenetic distance between these two species [46]. By contrast the zoonotic species *P. knowlesi*, which has been recently adapted to culture in human red blood cells [47, 48], offers several advantages, including a much closer evolutionary relationship to *P. vivax* [46] and high rates of transfection [47]. One of the lines adapted to *in vitro* culture in human erythrocytes, *P. knowlesi* strain A1-H.1, is derived from the strain A1 which was extracted from an American military surveyor in the 1960s [49] and is sensitive to all common antimalarials [47], making it the perfect genomic background to test for the acquisition of drug resistance.

We therefore investigated *P. knowlesi* as a model to systematically study *P. vivax* drug resistance-associated genes. *P. vivax* genes identified as being under recent evolutionary selection were expressed in *P. knowlesi*. In most cases two allelic variants of each gene were expressed, one from a clinical isolate obtained in an area where chloroquine resistance is limited, and the other from a reference genome generated from Indonesia, viewed as the epicentre of *P. vivax* chloroquine resistance. Studying the expression and localisation of these *P. vivax* proteins in *P. knowlesi*, and their impact on drug sensitivity, establishes *P. knowlesi* as a rapid and viable model for studying *P. vivax* biology.

## Materials and methods

### Construct generation

Constructs for episomal expression of *P. vivax* genes in *P. knowlesi* were constructed using the plasmid PkconGFP_ep_ as a backbone—kindly provided by Robert Moon [47]. Each construct contains an hDHFR cassette to allow for positive selection of transfectants using pyrimethamine. We used two different sources of genetic material for the generation of *P. vivax* sequences: (1) genomic DNA from a Cambodian clinical isolate used as a template for PCR amplification, and (2) synthesised DNA encoding the protein sequences of the genomic reference strain P01 from Papua Indonesia [50].

*P. vivax* genomic material had been previously collected and was not generated for this study. In both cases, informed consent was obtained and the work overseen by the appropriate local ethical review bodies. For the PvP01 genome, used as a source of sequence of Indonesian *P. vivax* sequences, full details are provided in the publication describing that reference genome [50]. For the Cambodian sample, the clinical protocol was approved by the National Ethics Committee for Health Research in Cambodia and the NIAID Institutional Review Board in the United States (ClinicalTrials.gov identifier NCT00663546), with informed consent obtained from adult patients, and from the parents or guardians of child patients; this material is also previously referenced [42].

The drug resistance profiles of these isolates are not known as no parasites were ever isolated, only DNA. However, the locations from which they were collected have circulating *P. vivax* parasites with very different drug resistance profiles: PvP01 originated from Papua Indonesia, an area of high chloroquine resistance, while the Cambodian sequence was obtained from a location with significantly lower population levels of resistance [19, 38, 51, 52]. PvP01 sequences were synthesised based on reference sequences available on the PlasmoDB database (http://plasmodb.org/, [53]), the introns were excised and the coding sequence recodonised for human expression to increase the GC content and facilitate their synthesis. A 3xHA-tag was also added at the 3’ end of the recodonised Papuan sequences to enable protein localisation, whereas the Cambodian constructs generated by PCR were not tagged. In both cases, the genes were amplified using NEBuilder Hifi with KAPA HiFi with primers containing overhang sequences (forward primers sequence: 5’-TGCAGATCCCCGTAAAACCC-3’ -reverse primers sequence 5’-GCGGATATGGCAGCTTAATG-3’) and were then cloned into the backbone by Gibson assembly using the SmaI and SacII sites. This replaced the GFP sequence in PkconGFP_ep_ and placed the genes of interest under the PkHsp70 promoter sequence; meaning that the transcripts should be overexpressed stably throughout the whole asexual cycle.

### Parasite culture and transfection

*P. knowlesi* clone A1-H.1 was maintained and transfected as in Moon et al. [47]. Briefly, parasites were maintained in fresh human blood (obtained from NHS Blood and Transplant) at 2% haematocrit in complete medium (RPMI-1640, supplemented with 2.3 g/L sodium bicarbonate, 4 g/L dextrose, 0.05 g/L hypoxanthine, 2.5 mM HEPES, 0.5% Albumax II, 10% (v/v) Life Technologies heat-inactivated horse serum, 2 mM L-Glutamine, 25 mg/L gentamicin). They were tightly synchronised one growth cycle prior to transfection. Tightly synchronised schizonts were purified via centrifugation on a 55% Nycodenz gradient and mixed with 40 μg of DNA in P3 solution. Electroporation was performed with a Lonza 4D electroporator using programme FP158. After 30min in recovery media (23% haematocrit in complete medium), they were transferred to 5 mL of complete medium. The medium was changed to selection medium (0.1 nM pyrimethamine in complete medium) 24 h after the transfection. Parasites were subsequently maintained in selection medium at all times. Step by step protocols for culture, synchronisation and transfection have been submitted to protocols.io to provide additional detail [54–56].

### Genotyping

Around 100 μL of packed red blood cells was lysed with 0.15% saponin for 2 min. The pellet was washed in PBS, then DNA extraction was performed using the NucleoSpin Tissue kit (Macherey-Nagel). Short-amplicon PCR was performed to confirm the presence of the expected construct and rule out cross-contamination, and plasmid copy number was assessed by qPCR (SsoAdvanced Universal SYBR Green Supermix, BioRad). qPCR was performed using primers binding to the promoter controlling the genes of interest expression (PkHsp70F and PkHsp70R, Table 1), to allow the same primers to be used on all lines. Plasmo1 and Plasmo2 primers amplifying *P. knowlesi* 18S rRNA [57] (Table 1) were used as a reference. The average number of plasmids was determined from the threshold cycle (Ct) values using ΔΔCt method.

**Table 1:** List of primers used in quantitative PCR.

Purpose	Primers name	Sequences 5’-3’
qPCR primers	PkHsp70F	GCGCTGGGGAGTACACATAT
	PkHsp70R	GGGACAATACGCACGATCCT
qPCR reference primers	Plasmo1	GTTAAGGGAGTGAAGACGATCAGA
	Plasmo2	AACCCAAAGACTTTGATTTCTCATAA

### Immunoblotting

Approximately 10 mL of cultures at 2% haematocrit mainly late stage *P. knowlesi* parasites was pelleted and washed once in PBS. The washed pellet was incubated in saponin 0.15% with protease inhibitor (1X, Roche) for 1 min on ice, which disrupts the erythrocyte membranes but leaves the parasitophorous vacuole membrane intact. Released parasites were pelleted and washed once in ice-cold PBS containing protease inhibitor. DNAse I (ThermoFisher) was added to the pellets and incubated for 30 min at 37°C. Parasites were resuspended in the same volume of Laemmli 2X (4% SDS, 20% Glycerol, 125 mM Tris-HCl pH 6.8, 10% β-mercaptoethanol, 0.02% bromophenol blue) and incubated for 45 min at 37°C, except for parasites expressing PVX_003935-HA-IND which were incubated at room temperature to avoid protein aggregation. Samples were then loaded onto NuPAGE 4-12% Bis-Tris Protein gels (Invitrogen) and run for 50 min at 200 V in MOPS buffer (Invitrogen). Proteins were transferred onto 0.45 μm nitrocellulose membranes (Amersham) for 1 h at 30 V, and the membranes were subsequently blocked in 5% milk/PBS. The primary antibody—rat α-HA (1:2000 Roche clone 3F10)—was added in 5% milk/PBS and incubated overnight at 4°C. The membranes were washed 3 times in PBS-Tween 0.1% (PBS-T) for 10 min. Goat α-rat-HRP antibody (1:40000 Abcam) was added in 5% milk/PBS and incubated for 1 h. The 3 washes were repeated and the membranes were imaged using the ECL Prime Western Blotting Detection Reagent kit (Amersham).

To establish whether the amount of protein was constant across samples, membranes were stripped and reprobed with an antibody detecting a housekeeping protein to act as a loading control. Membranes were incubated twice for 5 min in stripping buffer (0.2M glycine, 0.1% SDS, 0.1% Tween20, pH 2.2), then rinsed twice in PBS-T for 10 min and twice in PBS for 10 min. Membranes were then re-blocked in 5% milk/PBS overnight at 4°C. The primary loading control antibody—rabbit α-PfERD2 (1:2000 MR4)—was added in 5% milk/PBS for 1h. The membranes were washed 3 times in PBS-T for 10 min and incubated with goat α-rabbit-HRP antibody (1:40000 Abcam) in 5% milk/PBS for 45 min. After repeating the 3 washes, the membranes were imaged as before. For more details on the procedure, see the protocol on protocols.io [58].

### Immunofluorescence assays

To localise expressed proteins incorporating the HA tag, immunofluorescence assays were performed using methodology set out in Tonkin et al. [59], with minor modifications. When indicated, cells were incubated with 20 nM MitoTracker CMXRos (Sigma) for 30 min at 37°C prior to fixation. Cells were washed once in DPBS before fixing in a solution 4% paraformaldehyde, 0.0075% glutaraldehyde in DPBS. After one wash in DPBS, they were permeabilised in 0.1% Triton X-100/DPBS. Another wash in DPBS was performed, and cells were blocked in 3% BSA/DPBS overnight at 4°C. Primary antibodies diluted in 3% BSA/DPBS were added and incubated for 1 h. Dilutions and antibodies used were as follows: rat α-HA 3F10 (Roche, 1:500), rabbit α-HA (Abcam, 1:500), rabbit α-PfERD2 (MR4, 1:500), rat α-PfAMA1 (MR4, 1:200), rat α-PfBIP (MR4, 1:200), rabbit α-LDH (1:2000). After antibody incubation, cells were washed 3 times in DPBS for 10 min; and the secondary antibodies were added diluted 1:5000 in 3% BSA/DPBS and incubated for 1h. Cells were then washed 3 times in DPBS for 10 min and mounted with ProLong Gold Antifade Mountant with DAPI (ThermoFisher). The slides were sealed and imaged on a DMi8 (Leica Microsystem) using LasX software. Images were processed using ImageJ 1.51k software [60]. Our protocol is available on protocols.io [61].

### SYBRGreen assay

Chloroquine diphosphate and mefloquine hydrochloride were purchased from Sigma-Aldrich and dissolved in distilled water and DMSO respectively. Growth inhibition assays were performed as described by van Schalkwyk et al. [62] with the following modifications. Serial drug dilutions were prepared in triplicates in 100 μL of complete media. A drug-free control row was included in all assays to act as a negative control. Parasites were broadly synchronised, and 100 μL of culture containing >90% ring stage parasites was added to the drug dilutions to generate a final haematocrit and parasitaemia of 2% and 0.5% respectively. The last row of wells on each plate was seeded with red blood cells only to act as a fluorescence control. Plates were incubated at 37°C in normal culture conditions for 1.5 growth cycles (42 h). Cells were lysed by adding 100 μL of lysis buffer (20 mMTris, 5 mM EDTA, 0.008% (w/v) saponin, 0.08% (v/v) Triton X-100, pH 7.5) containing 3X of SYBR Green I (Invitrogen). Fluorescence was read on a FLUOstar Omega plate reader (BMG Labtech) at 490 nm excitation and 520 nm emission after 15 min incubation. Our step-by-step experimental protocol is available on protocols.io [63]. We performed the drug assays in 3 to 4 biological replicates and calculated the IC_50_ using the drm function from the drc library [64]. Statistical comparisons were performed using one-way ANOVA test with Holm’s multiple comparison tests (R 3.5.0 [65]).

## Results

### Selection of *Plasmodium vivax* genes previously implicated in drug resistance

Several recent population genetic analyses of *P. vivax* have identified genes under positive selection pressure [42, 66–69]. In *P. falciparum*, the most highly positively selected genes within populations are frequently those associated with drug resistance, such as *pfcrt* and *pfmdr1* [41]. Positively selected genes in *P. vivax* have also been hypothesised to be involved in drug resistance, although experimental proof is lacking due to the technical difficulties of working with *P. vivax*. We, therefore, sought to investigate the use of *P. knowlesi* as a model system to functionally test the impact of *P. vivax* gene variants on drug responses.

The study by Pearson et al. [42] sequenced more than 200 *P. vivax* clinical samples, primarily from Western Thailand, Western Cambodia and Papua Indonesia; chloroquine resistance is a particular concern in Papua Indonesia. We selected genes for expression based on the population genetic data from this study, focusing on both positive selection (a hallmark of drug resistance genes) and also population differences (e.g. genes under selection in regions with high drug resistance, but not in regions with low drug resistance). Four genes —PVX_101445, *pvdhfr*, *pvdhps*, PVX_084940—were selected because they had the highest scores in the cross-population extended-haplotype homozygosity test (XP-EHH) and so appeared to be under strong positive selection everywhere, regardless of local drug resistance profiles. Three genes —PVX_122995, PVX_003935, *pvap2-l* (PVX_081810)—were selected because they contained SNPs with high fixation index in one or several studied regions (FST >0.9), meaning they are under strong selection in some populations and not others; in two cases the SNPs are found only in Papua Indonesia, where there is high levels of chloroquine resistance. We also focused on these three genes because they encoded features such as multi-membrane spanning regions that are a hallmark of transporters, and are therefore strong drug resistance candidates [70]. Another four genes that would have fallen into these categories, PVX_002905, PVX_079910, *pvcrmp1* and *pvcrmp3*, were omitted because their size (>9 kb) prevented us from making transfection constructs. We also attempted to generate a construct for *pvmrp1*, another candidate with a high XP-EHH score, but were unable amplify it, possibly due to its large size and low GC content, leading us to exclude this gene as well. Finally, we added *pvmdr1*, which has a highly differentiated duplication in Western Thailand and has previously been implicated in both mefloquine and chloroquine resistance in *P. vivax* [19, 33, 35, 38], although as noted above, some of the clinical data is contradictory. The *P. vivax* ortholog of the main chloroquine resistance gene in *P. falciparum*, *pvcrt*, was not included as no signal of selective pressure has been found on this gene in several studies [42, 66, 67, 69], and the gene has previously been thought to be not related to chloroquine resistance in *P. vivax* [20, 21, 36], although again, this is a topic of some dispute. In total, 8 genes were considered as candidates for *P. vivax* drug resistance markers and selected for expression in *P. knowlesi* (Table 2, S1 Fig).

**Table 2:** *Plasmodium vivax* genes expressed in *Plasmodium knowlesi*.

Gene ID Gene name	Gene length	Properties	Population genetic features [30]
PVX_101445	989	conserved *Plasmodium* protein Transmembrane protein Dispensable in *P. falciparum* [71]	Very strong signature of selection in PID XP-EHH WTH vs PID: 2.2 × 10^−18^ Highly differentiated duplication in PID Fixed SNP (I81L, R97G) in PID
PVX_122995 *pvdmt1*	1311	Putative transporter Transmembrane protein Dispensable in *P. falciparum* [71]	Highly differentiated SNP (V217I) in WTH and WKH
PVX_003935	4343	Putative amine transporter Transmembrane protein Essential in *P. falciparum* [71]	Fixed SNP (G932S) in PID
PVX_084940	1753	Putative voltage-dependent anion-selective channel protein Essential in *P. berghei* [72]	Strong signature of selection in WTH and WKH XP-EHH WTH vs WKH: 5.6 × 10^−15^
PVX_089950 *pvdhfr*	1875	Dihydrofolate reductase SNPs implicated in pyrimethamine resistance in *P. vivax* [30]	Strong signature of selection in WTH XP-EHH WTH vs WKH: 1.7 × 10^−11^
PVX_080100 *pvmdr1*	4395	ABC transporter SNPs and CNV correlated with chloroquine and mefloquine resistance in *P. vivax* [19, 35, 38]	Highly differentiated duplications in WTH
PVX_123230 *pvdhps*	2549	Dihydropteroate synthetase SNPs implicated in sulfadoxine resistance in *P. vivax* [73]	Strong signature of selection in WTH XP-EHH WTH vs WKH: 8.2 × 10^−11^
PVX_081810 *pvap2-l*	3951	Transcription factor with AP2 domain(s) Involved in activation of liver stage genes in *P. berghei* [74]	Highly differentiated SNP (M862V) in PID

PID: Papua Indonesia (high rates of chloroquine resistance), WKH: Western Cambodia (low rates of chloroquine resistance), WTH: Western Thailand (low rates of chloroquine resistance, emergence of mefloquine resistance).

### Generation of *Plasmodium knowlesi* lines expressing *Plasmodium vivax* proteins

Chloroquine resistance in particular is thought to be at high prevalence in Indonesia, and the molecular mechanism of *P. vivax* chloroquine resistance is unclear and disputed. We therefore generated two versions of each gene for expression, one based on the genome sequence of a Cambodian *P. vivax* clinical isolate (CMB), a region where *P. vivax* chloroquine resistance is thought to be mostly absent, and one based on an Indonesian reference genome sequence (IND), where *P. vivax* chloroquine resistance is widespread. As described in the Methods, CMB alleles were generated by amplifying directly from the genomic DNA of a Cambodian *P. vivax* clinical isolate. IND alleles were synthesized (GeneArt Strings, ThermoFisher), with sequences based on the published PvP01 genome [50]. All IND alleles had 3 copies of the HA epitope tag added at the 3’ end to allow detection of expression by immunoblot and localisation using immunofluorescence. For genes *pvap2-l* and PVX_084940 only the CMB or HA-IND versions could be generated respectively. PVX_084940 could not be amplified from the Cambodian isolate genome despite several attempts (perhaps due to sequence variability in the primer binding sites), and *pvap2-l* is a long intronless gene which was considered by commercial companies too difficult to synthesise (due to the presence of repetitive sequences). In total therefore, 14 constructs were generated: both CMB and HA-IND variants of six genes (PVX_003935, PVX_101445, PVX_122995, *pvdhfr*, *pvdhps*, and *pvmdr1*) and only a single variant of two others (*pvap2-l*-CMB, PVX_084940-HA-IND). Several highly differentiated SNPs highlighted by the Pearson et al. study are present in the constructs generated (S2 Table). All 14 constructs were inserted into an expression vector backbone which placed them under the control of the strong, constitutive *P. knowlesi* hsp70 promoter, and successfully transfected into *P. knowlesi* parasites. All produced stable pyrimethamine-resistant isolates, with transfectants typically observed within the week following transfection, and showing high parasitaemia leading to expansion around day 14, emphasising the high transfection efficiency of the system [47]. Transfected strains were genotyped using short amplicon qPCR, and the presence of episomes was confirmed before further characterisation.

**Fig 1:**
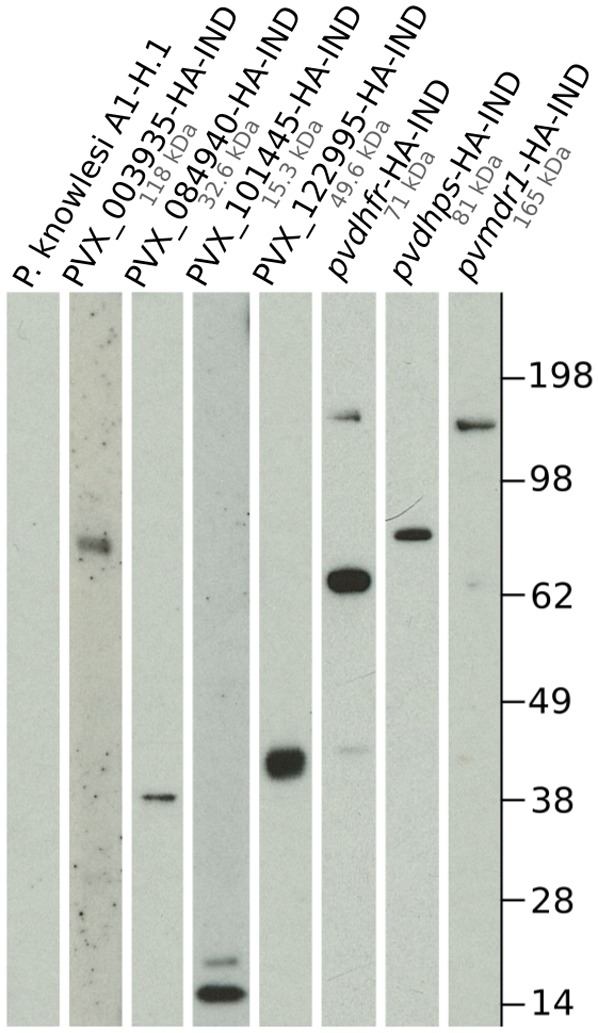
Confirmation of -HA-IND allele protein expression by immunoblotting. Parasite lysates were prepared and run in denaturing conditions as described. Proteins were detected with an α-HA antibody. A lysate of the parent *P. knowlesi* strain A1-H.1 was used as a negative control.

To confirm protein expression we performed immunoblotting on the lines expressing -HA-IND alleles, using antibodies that recognise the C-terminal HA-tag. Protein extractions were performed on broadly synchronous cultures containing primarily late stage parasites and immunoblotting performed as described in Methods. As shown in Fig 1, all the *P. vivax* proteins could be readily detected in *P. knowlesi* extracts.

The majority of proteins detected had molecular weights similar to that predicted by their primary amino acid sequence. PVX_003935 and PVX_122995 proteins ran slightly smaller than their expected sizes, 90 kDa and 40 kDa instead of 118 kDa and 49 kDa respectively, but both contain hydrophobic regions predicted to form transmembrane domains, which can often affect mobility in SDS-PAGE. PVX_003935 showed the weakest signal, and could only be detected when parasite lysates were kept at room temperature, rather than incubated at 37°C as for other lysates. PVX_003935 has high levels of hydrophobicity and multiple predicted transmembrane domains, and aggregation of such proteins after denaturation, particularly at high temperatures, is a common problem.

We observed a faint higher molecular weight secondary band at 143 kDa in PvDHFR-HA-IND expressing parasites, at approximately double the predicted size of PvDHFR. This band was present when the samples were not heated or heated at 37°C but was not visible if the samples were boiled at 85°C (S2A and S2A Fig). This finding suggests that the band is the result of dimerisation of PvDHFR which has also previously been observed for PfDHFR [50]. A faint higher molecular weight secondary band was also observed for PVX_101445, but it was much closer to the predicted size, and might be the result of post-translational modification. We also detected a smaller secondary band at around 70 kDa in the line expressing PvMDR1-HA-IND when increasing the exposure time (S2C Fig). This could be a cleavage product, perhaps between the two 6-transmembrane domain channels, based on the observed size. A similar sized secondary product for PfMDR1 was also observed by Elandaloussi and colleagues [75] in *P. falciparum* extracts, and by van Es and colleagues [76] while overexpressing PfMDR1 in CHO cells, suggesting that the two MDR1 homologues may be proteolytically cleaved in a similar way.

Overall all seven lines transfected with HA-tagged *P. vivax* constructs showed clear expression of *P. vivax* protein at near the expected sizes with no sign of notable degradation. This confirms *P. knowlesi* as a rapid and efficient system to express and study *P. vivax* proteins.

### Immunolocalisation of *Plasmodium vivax* proteins

**Fig 2:**
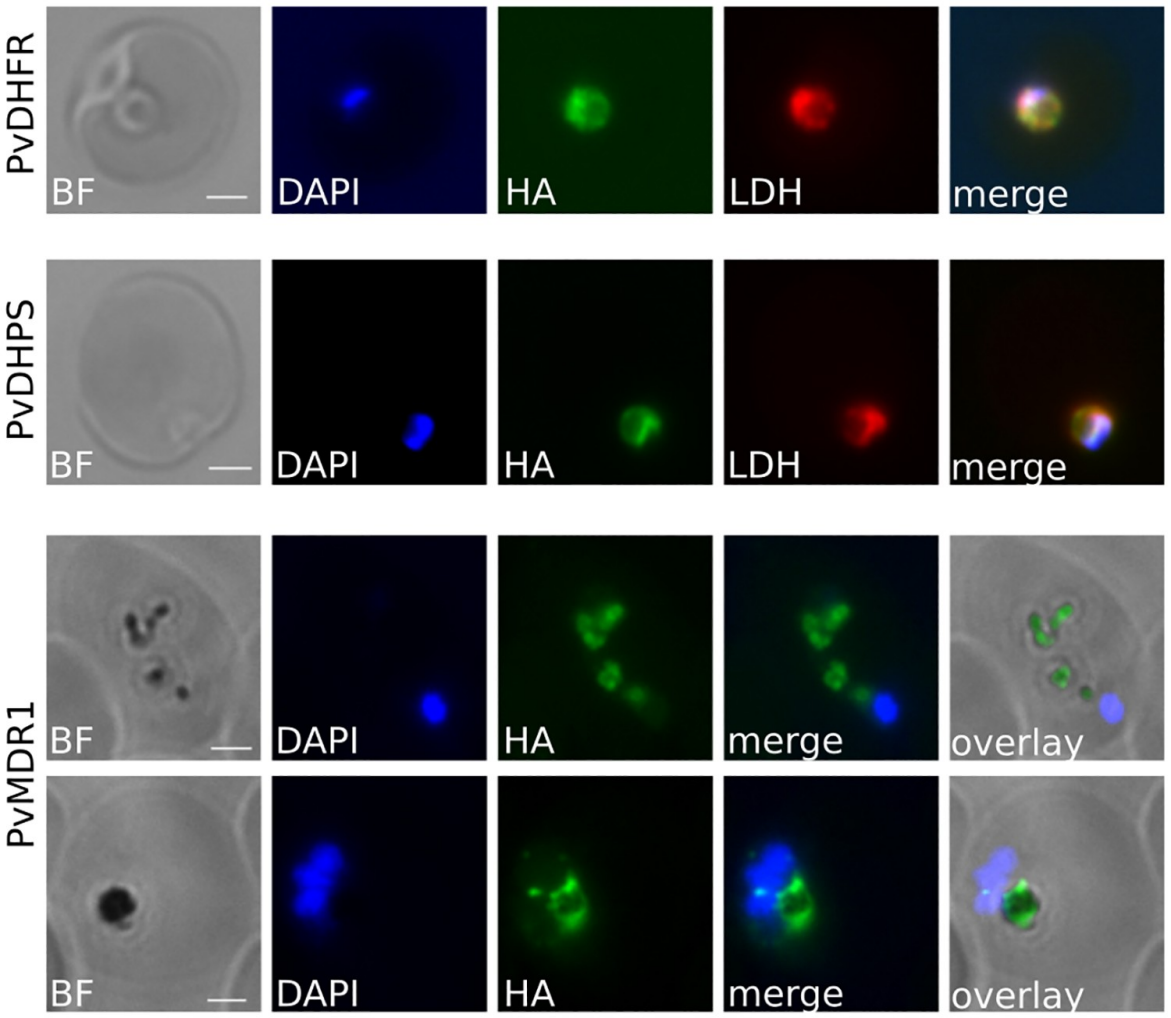
Localisation of PvMDR1, PvDHPS and PvDHFR in *P. knowlesi* by immunofluorescence. Epitope tagged *P. vivax* proteins were localised using a rat α-HA antibody and an AlexaFluor^®^ 488 secondary. LDH was imaged using rabbit α-PfLDH antibody and an AlexaFluor^®^ 680 secondary antibody. Scale bar 2 μm.

To identify the subcellular localisation of the -HA-IND alleles, we performed immunofluorescence assays using α-HA antibodies. We first started with the best-characterised proteins, PvMDR1, PvDHPS and PvDHFR. Although these three proteins have never been functionally studied in *P. vivax*, their *P. falciparum* homologues have been extensively investigated. PfMDR1 is known to localise to the food vacuole membrane [75, 77] and PfDHFR is a cytoplasmic enzyme [78]. PfDHPS has never been localised, although both cytosolic and mitochondrial localisation have been described for *Arabidopsis thaliana* homologues [79]. As shown in Fig 2, the *P. vivax* orthologs of PvDHFR and PvMDR1 seem to localise at the expected locations when expressed in *P. knowlesi*. PvMDR1-HA-IND staining was consistently associated with the dark bodies that are hemozoin crystals, located inside the food vacuole. PvMDR1-HA-IND staining appears to encircle the hemozoin, supporting the localisation of PvMDR1 to the food vacuole membrane. PvDHFR-HA-IND showed a similar pattern of staining to LDH, a cytosolic maker (Fig 2). Colocalisation with LDH was also observed for PvDHPS (Fig 2) which did not show similar localisation as MitoTracker CMXRos (S3 Fig), suggesting the enzyme is cytosolic rather than mitochondrial. All three *P. vivax* proteins therefore localised as predicted from the location of their homologues, in either *P. falciparum* or *A. thaliana*. This suggests that they are being trafficked correctly, despite being constitutively overexpressed in a heterologous parasite species.

**Fig 3:**
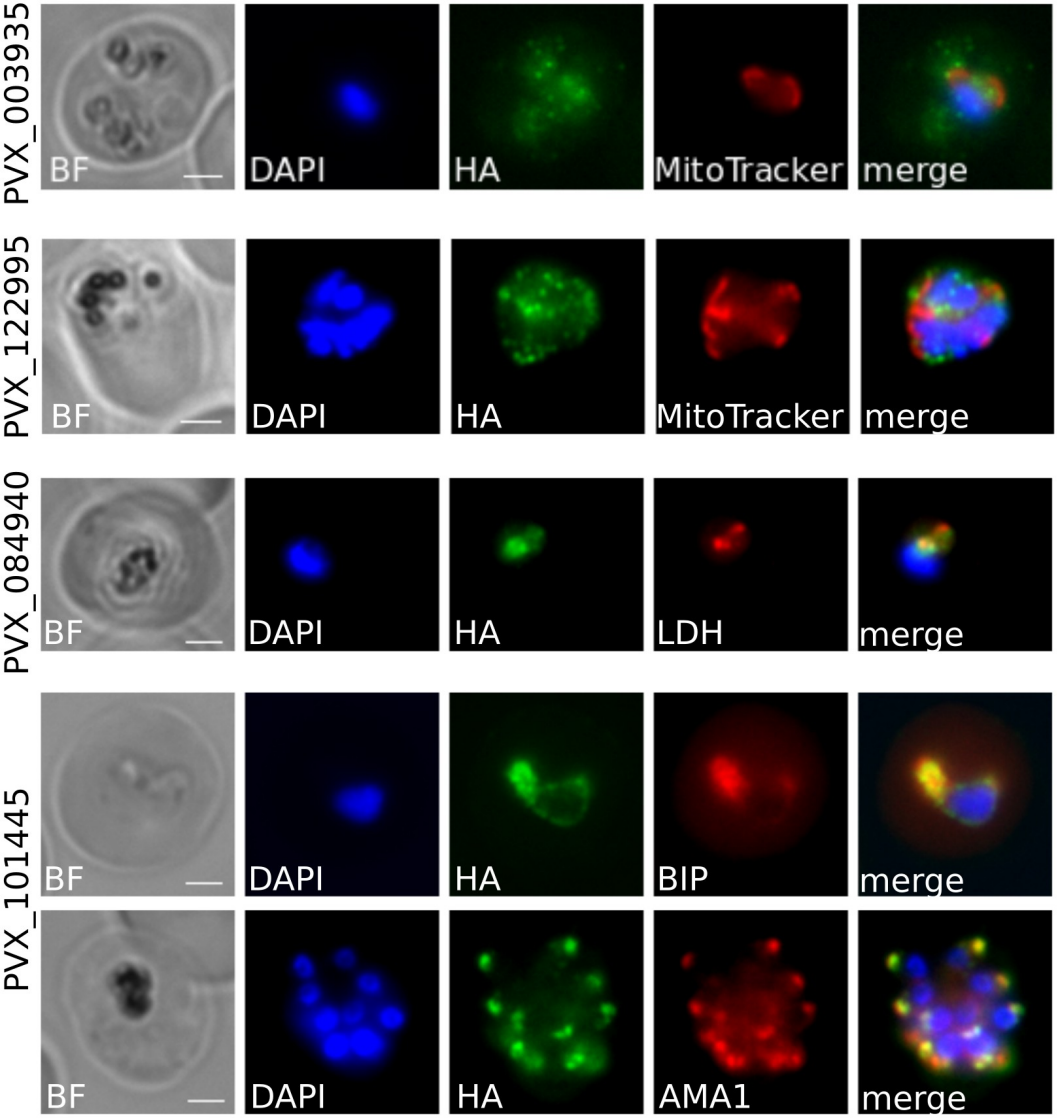
Localisation of PVX_101445, PVX_084940, PVX_122995 and PVX_003935 in *P. knowlesi* by immunofluorescence. *P. vivax* proteins were localised using either a rat α-HA antibody or a rabbit rat α-HA antibody. Both α-HA antibodies were conjugated with an AlexaFluor^®^ 488 secondary. Costaining markers AMA1, BiP and LDH were conjugated with an AlexaFluor^®^ 680 secondary antibody. MitoTracker CMXRos emits at 599 nm. Scale bar 2 μm.

We then attempted to localise the remaining proteins, which have never previously been studied in any *Plasmodium* species. PVX_084940 is predicted based on homology with eukaryotic porins to localise to the outer membrane of the mitochondria; we therefore used MitoTracker CMXRos which accumulates in the mitochondria lumen as a co-stain to investigate PVX_084940 protein localisation. As shown in Fig 3, PVX_084940-HA-IND staining was closely linked to, but not entirely coincident with, MitoTracker. In some cases PVX_084940-HA-IND seemed to surround the MitoTracker staining, which would correlate with its annotation, however this pattern was not consistently observed (S4 Fig). These data suggest that PVX_084940 is associated either with the mitochondrion or the intimately-associated apicoplast, for which no marker exists in *P. vivax*, and serves as a caution about predicting location based entirely on homology.

PVX_101445 showed different localisation depending on the stage of the parasite. In early stages, the protein colocalises with the chaperone BiP located in the endoplasmic reticulum suggesting PVX_101445 is processed and/or stored in this organelle (Fig 3). During trophozoites the localisation becomes harder to evaluate, perhaps representing an artefact of the overexpression inherent in our screening system, but a clear pattern is visible in late schizonts once the merozoites are formed. The protein has an apical localisation with a punctate pattern close to AMA1, a micronemal protein. Our observation suggests that, like some proteins involved in invasion such as MSP1, PVX_101445 is firstly expressed in the ER where it could undergo some post-translational modifications. It would then be exported to a secretory organelle such as the rhoptries or the micronemes when the merozoites are being formed. This finding could highlight a new protein involved in *Plasmodium* invasion and further investigation on PVX_101445 could be of interest.

Identifying a confirmed location for PVX_003935 and PVX_122995 proved more challenging. Both had distributed punctate distributions, and neither protein colocalised with markers in the cytoplasm (α-LDH), the mitochondria (MitoTracker CMXRos), the ER (α-BIP) or the cis-Golgi (α-ERD2) (Fig 3, S5 and S6 Figs). PVX_003935 labelling frequently seemed to have a punctate pattern that surrounds Erd-2 staining, a pattern reminiscent of the Golgi apparatus. In this case, it would have to be the trans-Golgi due to the lack of colocalisation with the cis-Golgi marker ERD2. We thus hypothesise that PVX_003935 might be a trans-Golgi protein or trafficked in the trans-Golgi but as no markers are available for this organelle in *Plasmodium* spp., this hypothesis could not be tested. Further confirmation of the localisation of these proteins was not possible due to the lack of markers for other *Plasmodium* blood-stage organelles.

### Drug sensitivity assays

**Fig 4:**
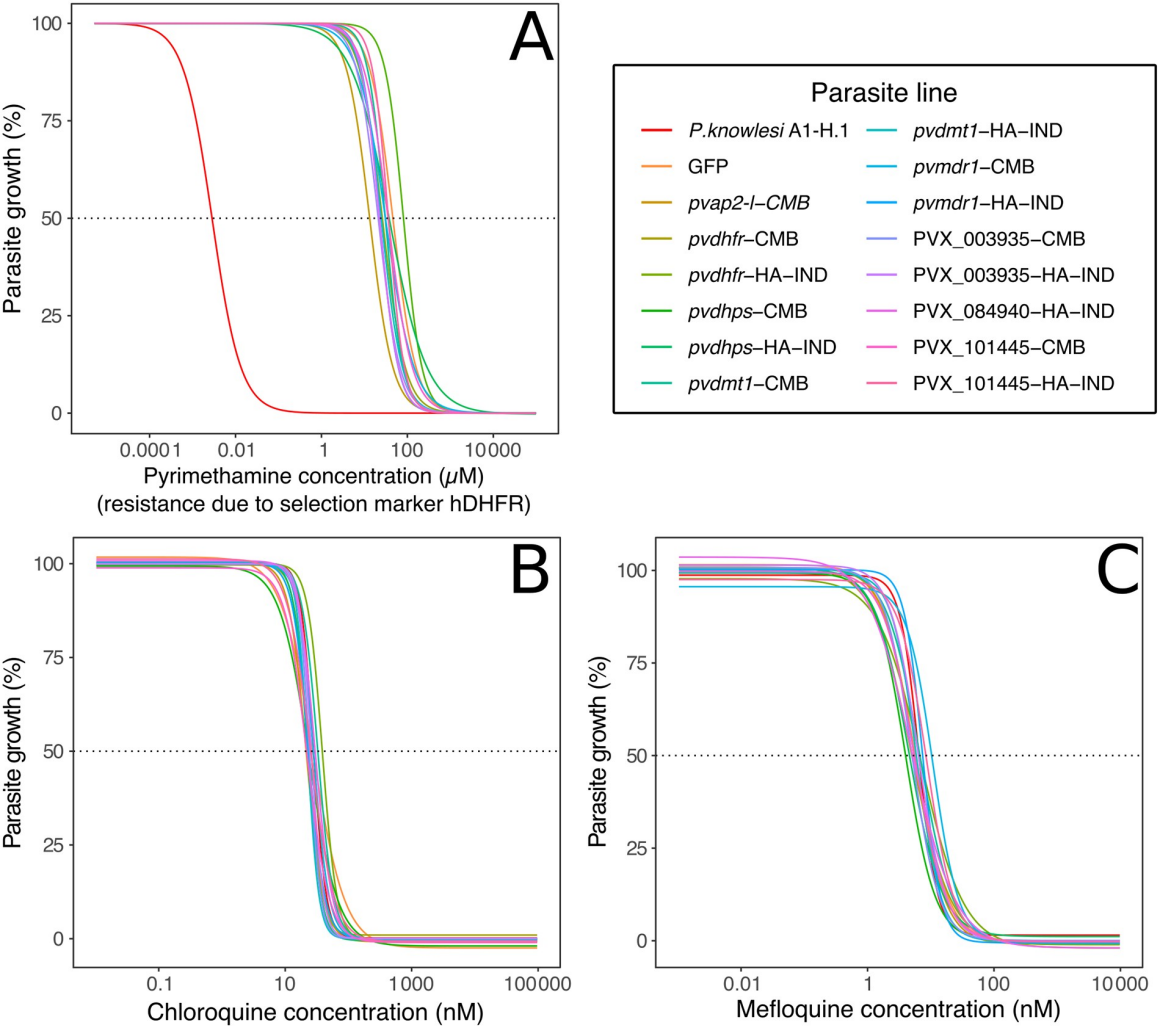
Transfected *P. knowlesi* lines show no evidence of a change in sensitivity to chloroquine or mefloquine but are resistant to pyrimethamine due to the expression of hDHFR. (A) Pyrimethamine dose-response curves. (B) Chloroquine dose-response curves. (C) Mefloquine dose-response curves.

Having established that all -HA-IND alleles expressed proteins of the expected size and in expected locations, where such locations could be predicted, we then assessed whether overexpression of either the -HA-IND or -CMB alleles of these *P. vivax* proteins had any impact on *P. knowlesi* sensitivity to common antimalarials. To do so, we adapted the method of van Schalkwyk et al. [62] and focused on chloroquine and mefloquine responses, as these comprise the majority of drug resistance reported in the field [13, 27, 29, 36]. As an initial control, we used this drug assay protocol to measure the IC_50_ of all the lines to pyrimethamine. Because all our constructs include human DHFR as a selective marker, we expected that only wild-type *P. knowlesi* strain A1-H.1 would be sensitive to this drug and that was indeed the pattern that was observed (Fig 4A). As a result we could not test antimalarials targeting the folate synthesis pathway, such as sulfadoxine and pyrimethamine, since the presence of hDHFR is likely to mask any effect of the overexpression of the gene of interest on sensitivity to these drugs. The clear and consistent signal of resistance shown in our pyrimethamine assay suggests that our approach has the power to detect changes in IC_50_ of the order of magnitude that are observed in *P. falciparum* chloroquine resistance.

We next performed chloroquine and mefloquine sensitivity assays on all 14 stable lines, as well as the wild-type strain, *P. knowlesi* strain A1-H.1 and a line transfected with the vector backbone only (expressing GFP and labelled as such) to act as negative controls. We generated dose-response curves for all lines with 3 to 4 biological replicates (Fig 4B and 4C). The 95% CI of the IC_50_ for all strains fell within a 2-fold window around *P. knowlesi* strain A1-H.1 (S7 Fig) indicating that there was no clear shift in drug sensitivity for any transfected lines. This was confirmed using a two-way ANOVA test and Holm’s method to adjust p-values. No significant difference was found between any transfected lines and either *P. knowlesi* strain A1-H.1 or the backbone GFP control. Drug sensitivity of the two different alleles (where available) was also compared but no significant variation was found in any case.

## Discussion

*P. vivax* drug resistance is of central importance and concern, as drug resistance has been linked to severe cases in Southeast Asia [80]. Resistance to almost all common antimalarials has been reported for *P. vivax* [1, 13, 18–20, 23, 27, 29–31, 36, 38, 81], with artemisinin-combined therapy being the only exception, at least for now. The fact that mefloquine resistance has been reported in several areas whereas it has never been recommended for *P. vivax* treatment [18, 29, 81] suggests that the drug pressure of mefloquine use for confirmed or supposed *P. falciparum* cases is strong enough on *P. vivax* to lead to the emergence of *P. vivax* drug resistance. We can therefore expect *P. vivax* drug resistance to continue to emerge, regardless of official treatment recommendations. Understanding *P. vivax* drug resistance is complicated by the difficulty of determining whether parasite reappearance comes from a new infection, recrudescence after treatment or from relapse due to hypnozoite activation [8]. Several recurrences have been observed after artemisinin-combined therapy [82–88] and in most cases the conclusion that these are relapses driven by hypnozoites have not had strong empirical support. This highlights a significant gap in our knowledge, and experimentally confirming molecular markers of *P. vivax* drug resistance would significantly improve and simplify drug resistance monitoring. It would allow better case management in areas where resistance is on the rise, helping preventing drug resistance spread. Confirming and validating such markers requires detailed experimental methods, but the lack of a *P. vivax* *in vitro* culture system in which genetic modification is possible is a significant roadblock for the field.

In this study we investigated the use of a new model, *P. knowlesi* strain A1-H.1, to express *P. vivax* proteins and study their implication in drug susceptibility. We were able to generate 14 lines expressing *P. vivax* proteins, and parasites were recovered quickly after transfection, emphasising the high efficiency of *P. knowlesi* transfection. Immunoblotting confirmed protein expression for all tagged lines with no notable degradation. *P. vivax* proteins seemed to be trafficked correctly; PvMDR1 localised around the hemozoin, suggesting it localises in the food vacuole membrane like PfMDR1, and both PvDHFR and PvDHPS enzymes colocalised with the cytosolic enzyme LDH. This is to our knowledge the first experimental confirmation of localisation for any of these highly studied and discussed *P. vivax* proteins, albeit in a setting of ectopic overexpression which could potentially induce mislocalisation. We also explored localisation of four previously unstudied *P. vivax* proteins. We found that PVX_101445 had an apical localisation in *P. knowlesi* schizonts which could suggest a role in invasion and that PVX_084940 consistently localised near to the mitochondrion, but not completely so, suggesting it could also be localised to the apicoplast. We hypothesised that PVX_003935 could be localised within, or trafficked by, the trans-Golgi, while PVX_122995 localisation could not be conjectured from our observations due to a lack of marker proteins to compare it with. Future work using super-resolution microscopy approaches might shed further light on the localisation of some of these proteins. These data clearly establish the validity of *P. knowlesi* for study of *P. vivax* proteins, including medium scale expression and localisation. Furthermore, these localisations provide insight into the biology of these candidate proteins, which may be particularly useful if in the future there is a need to understand the mechanisms by which these proteins act.

Our IC_50_ results for wild-type *P. knowlesi* were consistent with van Schalkwyk et al. [62] and did not indicate that *P. knowlesi* is especially resistant to mefloquine as previously reported [89] (this resistance might be limited to the simian W1 strain). All lines showed a sensitivity similar to wild-type for both chloroquine and mefloquine regardless of whether Cambodian or Indonesian allelic variants were expressed, suggesting episomal overexpression of either allelic variant of these proteins does not affect *P. knowlesi* drug susceptibility. At first glance therefore, our data suggests that these genes do not play a role in either chloroquine or mefloquine resistance. However, it is important to note that while these data do not support such a role, they in no way rule it out, as there are several possible technical explanations for the lack of a resistance phenotype. One complicating factor is that the isolates that were the source of the genomes used to create our transfection constructs were not assayed for drug resistance or sensitivity when they were collected, as part of that previously published work. Therefore despite the high reported level of resistance in Indonesia and the presence in our expressed sequences of highly differentiated SNPs that have been associated with drug resistance, the resistance or susceptibility of these isolates is unknown. A future study using genomic sequences from a sample with a well-characterised resistance phenotype would be valuable, although it should be noted that high quality *ex vivo* drug resistance assays for *P. vivax* are still largely in development rather than in widespread use. There are other technical considerations. The *P. vivax* genes were expressed from episomes on a wild-type *P. knowlesi* background, meaning that in each line the endogenous *P. knowlesi* homologue was still expressed. This could potentially exert a dominant effect and mask any phenotypic change induced by the *P. vivax* gene. In addition, the genes were not under the control of their native promoter, and were instead controlled by a strong promoter that drives expression throughout the intraerythrocytic life cycle. This approach has some advantages as it makes it much easier to generate multiple constructs, avoids the issues of estimating where native promoters start and end, and might also reveal over-expression phenotypes that would require gene duplication *in vivo*. However, this overexpression could fail to reveal a phenotype that requires finer tuning of expression, and if resistance were identified an additional control overexpressing the native *P. knowlesi* gene would be required to further explore the cause of resistance, and contribution, if any, of natural variants within the expressed *P. vivax* genes. This expression system was chosen in part because of technical limitations. The range of genetic tools available for *P. knowlesi* is still relatively limited, given the recent adaptation to culture in human blood [47, 48]. CRISPR/Cas9, now widely used in *P. falciparum*, was not in use in *P. knowlesi* at the time of the study.

This technology has recently been adapted to *P. knowlesi* [90], and should allow drug resistance candidates to be investigated by inserting *P. vivax* genes directly into the orthologous *P. knowlesi* locus, thereby eliminating any issues caused by expression of wild-type *P. knowlesi* genes in the background of these lines, ensuring a consistent copy number, and, in the case of essential genes, proving functionality of the heterologously expressed gene. Furthermore with this approach, the selective marker could be removed by recycling under negative selection [90], allowing sensitivity to all drugs to be assessed and avoiding the masking effect that existed for antifolate drugs in our study, which meant that we were unable to test for the effect of expressing the candidate genes on sensitivity to pyrimethamine and sulfadoxine. This approach would have the further advantage that genes of interest would be expressed under their native promoters, avoiding any issues associated with misregulation of transcription. The *hsp70* promoter which we used in this study provides relatively consistent transcription throughout the parasite lifecycle (according to evidence from the *P. falciparum* orthologue [91]), with a peak of expression in trophozoites at 18 hours post-invasion. This is similar to the time of peak transcriptional activity for the orthologues of PVX_123230, PVX_084940 and PVX_122995. Other candidate genes peak either earlier in the intra-erythrocytic cycle (PVX_101445, PVX_122995, PVX_080100) or later (PVX_089950, PVX_003935). These differences might also contribute to the absence of resistance observed, although the *hsp70* promoter still provides considerable transcription at these timepoints.

To our knowledge, only one other study has used an *in vitro* model to experimentally investigate *P. vivax* drug resistance markers. Auliff et al. (2010) [30], used *P. falciparum* as a model for studying the influence of *P. vivax* dihydrofolate-reductase on antifolate sensitivity. They adopted a similar episomal expression strategy and could show that mutations in *pvdhfr* lead to resistance to pyrimethamine, cycloguanil, WR99210 and clociguanil. While these data show that *P. falciparum* can be used to investigate *P. vivax* drug-resistance genes in the case of enzymes like DHFR, there are a number of reasons to suspect that *P. knowlesi* may be an even more effective model. *P. falciparum* is a member of the *Laverania*, a group of malaria parasites that diverged from other mammalian-infecting *Plasmodium* spp. early in evolutionary history—even the rodent malaria parasites *P. berghei*, *P. yoelii* and *P. chabaudi* are closer relatives of *P. vivax* than is *P. falciparum* [46]. By contrast, *P. knowlesi* is much more closely related to *P. vivax*. As a result, there are 278 genes, for instance, common to *P. vivax* and *P. knowlesi* but absent in *P. falciparum*, and both species lack *P. falciparum*’s extremely high AT-content. All these features make *P. knowlesi* an attractive model: if a potential drug resistance protein interacts with other proteins it is much more likely that these interactions will be conserved in *P. knowlesi* than in *P. falciparum*. In the case of a simple enzyme like DHFR, such concerns are not relevant, and even a model like yeast [92] can give good results, but it is unlikely that all drug-resistance genes fall into this category.

Further to these points of model-appropriateness, there are also practical considerations. *P. knowlesi* is far more amenable to transfection than is *P. falciparum*, achieving transfection efficiencies which are higher by an order of magnitude [47, 93, 94], offering broad possibilities as an experimental system [93–95]. The adapted strains can still infect the natural host of *P. knowlesi*, *Macaca fascicularis*, allowing studies to transition from purely *in vitro* to *in vivo* studies of pathology. In addition to being used as a model for other species, understanding *P. knowlesi* biology and the chain of events that lead to the adaptation of the parasite to human is also of great interest. To our knowledge, this is the first study using human blood adapted *P. knowlesi* as an *in vitro* model system to study *P. vivax* genes of unknown function. Our results are encouraging, showing high transfection yield and rapid generation of *P. knowlesi* transfectants as well as little degradation of *P. vivax* proteins and trafficking to the correct organelles, at least where such localisations are possible to predict. Further advances in *P. knowlesi* tools will make this model even more powerful for understanding the mechanisms of drug resistance in *P. vivax*.

## Supporting information

S1 Fig Candidate selection and cloning lead to the generation of 14 transfected *P. knowlesi* lines for further phenotyping. (TIF)

S2 Fig Additional immunoblots. (A) Parasite lysates were prepared and run in denaturing conditions as described. Proteins were detected with an α-HA antibody and a house-keeping gene, ERD2, was used as a loading control for all samples. (B) Parasites lysates were boiled at 80 degrees to enhance denaturation before running on SDS-PAGE gel and detected with an α-HA antibody. (C) PvMDR1 was detected with an α-HA antibody. Two main bands can be observed, the strongest one located around the expected size of 165kDa (white arrow) and a secondary product around 70kDa (black arrow).
(TIF)

S3 Fig Colocalisation of PvDHPS with a mitochondrial or cytosolic marker in *P. knowlesi* by immunofluorescence. PvDHPS was localised using a rat α-HA antibody and an AlexaFluor^®^ 488 secondary. LDH was imaged using rabbit α-PfLDH antibody and an AlexaFluor^®^ 680 secondary antibody. MitoTracker CMXRos emits at 599nm. Scale bar 2 μm.
(TIF)

S4 Fig Colocalisation of PVX_084940 with a mitochondrial or cis-Golgi marker in *P. knowlesi* by immunofluorescence. PVX_084940 was localised using a rat α-HA antibody and an AlexaFluor^®^ 488 secondary. ERD2 was imaged using rabbit α-PfERD2 antibody and an AlexaFluor^®^ 680 secondary antibody. MitoTracker CMXRos emits at 599nm. Scale bar 2 μm.
(TIF)

S5 Fig Colocalisation of PVX_003539 with several organelle markers in *P. knowlesi* by immunofluorescence. PVX_003539 was localised using either a rat or a rabbit α-HA antibody. Both α-HA antibodies were conjugated with an AlexaFluor^®^ 488 secondary. Costaining markers AMA1, ERD2 and LDH were conjugated with an AlexaFluor^®^ 680 secondary antibody. MitoTracker CMXRos emits at 599 nm. Scale bar 2 μm.
(TIF)

S6 Fig Colocalisation of PVX_122995 with several organelle markers in *P. knowlesi* by immunofluorescence. PVX_122995 was localised using either a rat or a rabbit α-HA antibody. Both α-HA antibodies were conjugated with an AlexaFluor^®^ 488 secondary. Costaining markers AMA1, BiP, ERD2 and LDH were conjugated with an AlexaFluor^®^ 680 secondary antibody. MitoTracker CMXRos emits at 599 nm. Scale bar 2 μm.
(TIF)

S7 Fig Parasite lines’ chloroquine (left dotplot) and mefloquine (right dotplot) IC_50_s calculated from 3 to 4 biological replicates. The error-bars represent the 95% confidence interval and a two fold window around the *P. knowlesi* strain A1-H.1 IC_50_ is indicated with dotted lines.
(TIF)

S1 Table Name and accession numbers of the genes and proteins mentioned in the article. (XLSX)

S2 Table Mutations present in the coding sequence of the Cambodian (-CMB) and Indonesian (-IND) gene sequences used in this study, compared to Sal1 reference(originally from El Salvador). SNPs in bold type are common to both the Cambodian and Indonesian alleles and SNPs in red were found to be highly differentiated in the Pearson et al. study to one population or the other [42].
(XLSX)
