# Supplementary figures and images for: Plasmodium knowlesi as a model system for characterising Plasmodium vivax drug resistance candidate genes
Source: PLoS Negl Trop Dis. 2019 Jun 3;13(6):e0007470. doi: 10.1371/journal.pntd.0007470 (PMC6564043; doi:10.1371/journal.pntd.0007470)

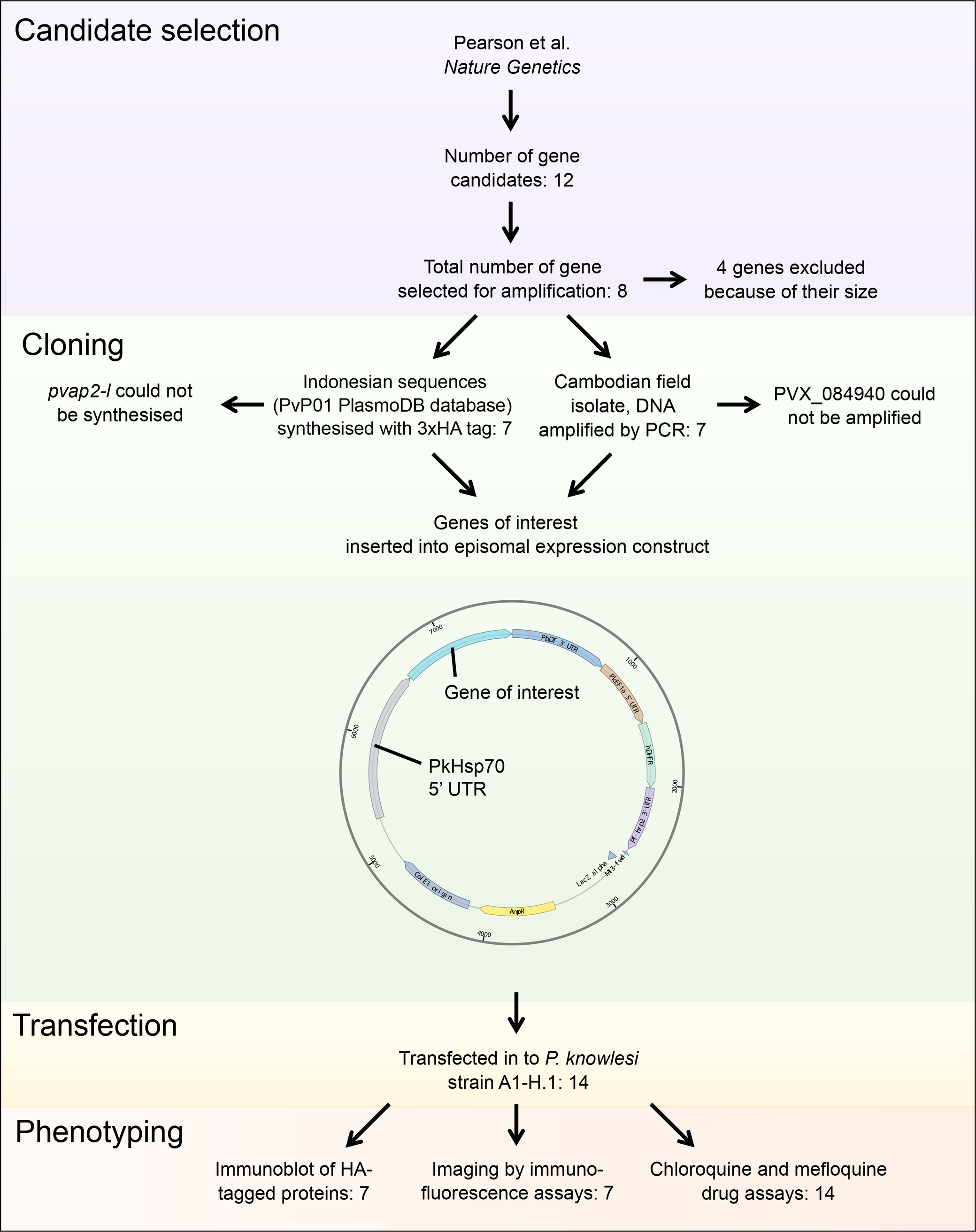

Supplement: S1 Fig — (TIF) [file pntd.0007470.s001.tif]

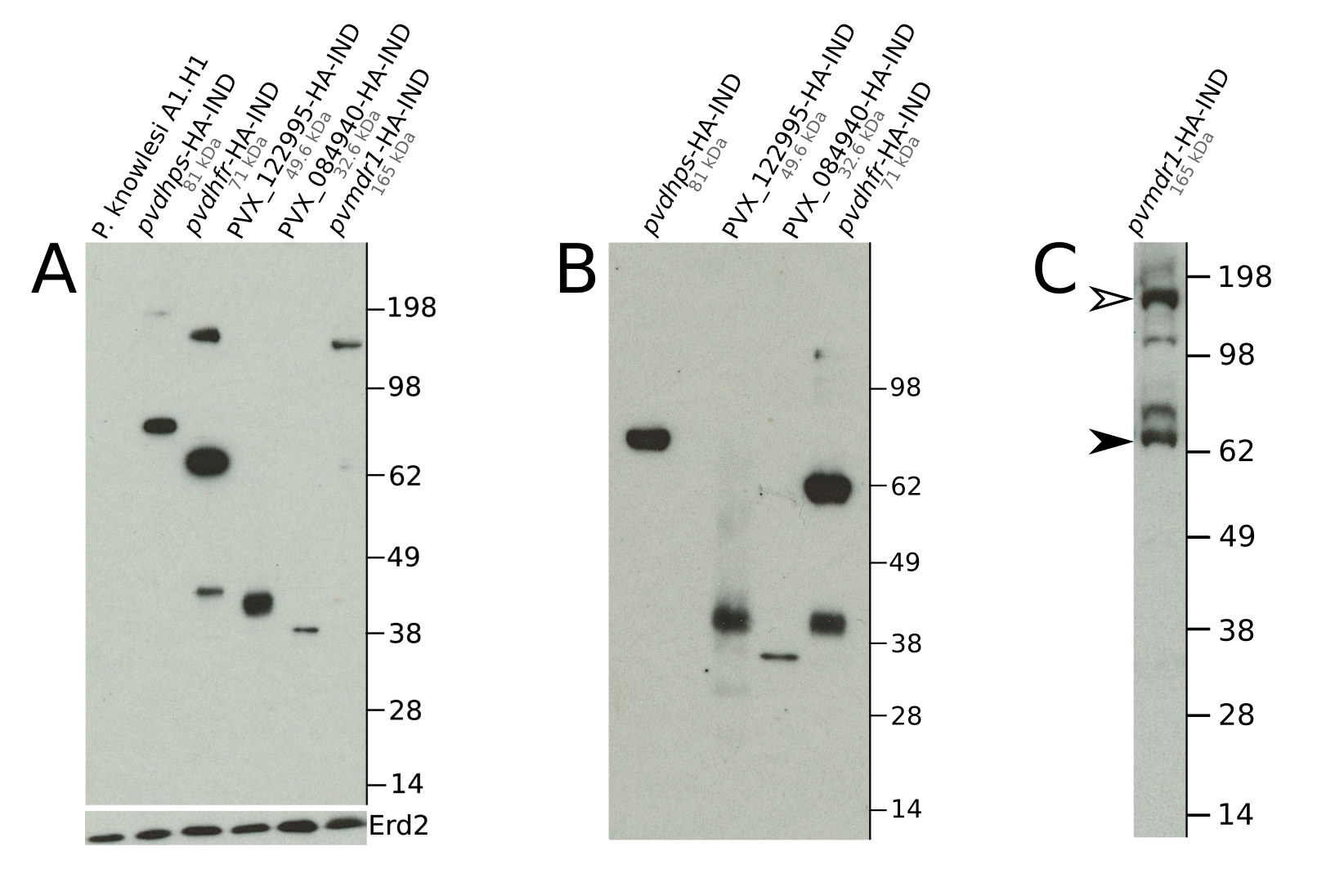

Supplement: S2 Fig — (A) Parasite lysates were prepared and run in denaturing conditions as described. Proteins were detected with an α-HA antibody and a house-keeping gene, ERD2, was used as a loading control for all samples. (B) Parasites lysates were boiled at 80 degrees to enhance denaturation before running on SDS-PAGE gel and detected with an α-HA antibody. (C) PvMDR1 was detected with an α-HA antibody. Two main bands can be observed, the strongest one located around the expected size of 165kDa (white arrow) and a secondary product around 70kDa (black arrow). (TIF) [file pntd.0007470.s002.tif]

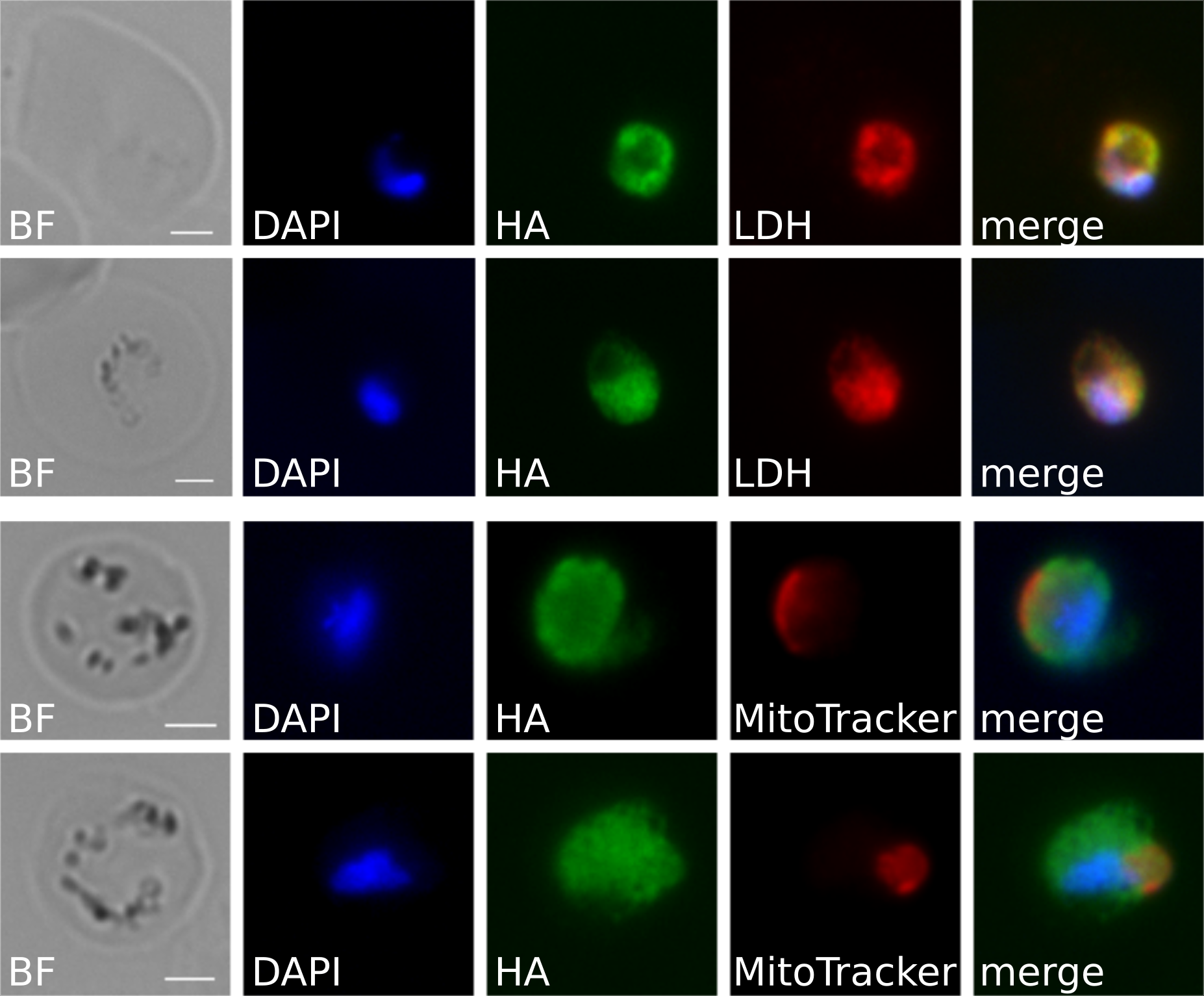

Supplement: S3 Fig — PvDHPS was localised using a rat α-HA antibody and an AlexaFluor® 488 secondary. LDH was imaged using rabbit α-PfLDH antibody and an AlexaFluor® 680 secondary antibody. MitoTracker CMXRos emits at 599nm. Scale bar 2 μm. (TIF) [file pntd.0007470.s003.tif]

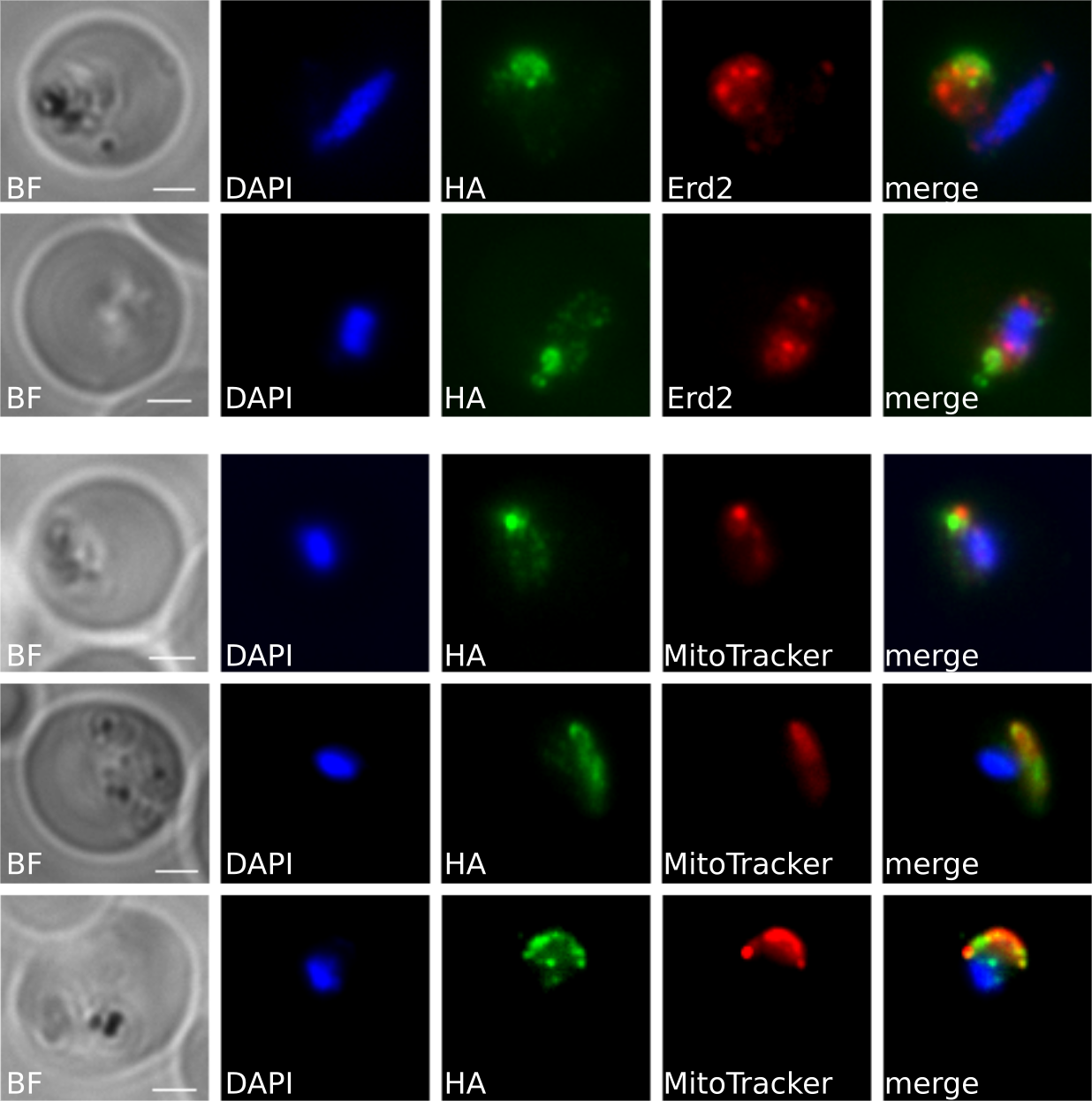

Supplement: S4 Fig — PVX_084940 was localised using a rat α-HA antibody and an AlexaFluor® 488 secondary. ERD2 was imaged using rabbit α-PfERD2 antibody and an AlexaFluor® 680 secondary antibody. MitoTracker CMXRos emits at 599nm. Scale bar 2 μm. (TIF) [file pntd.0007470.s004.tif]

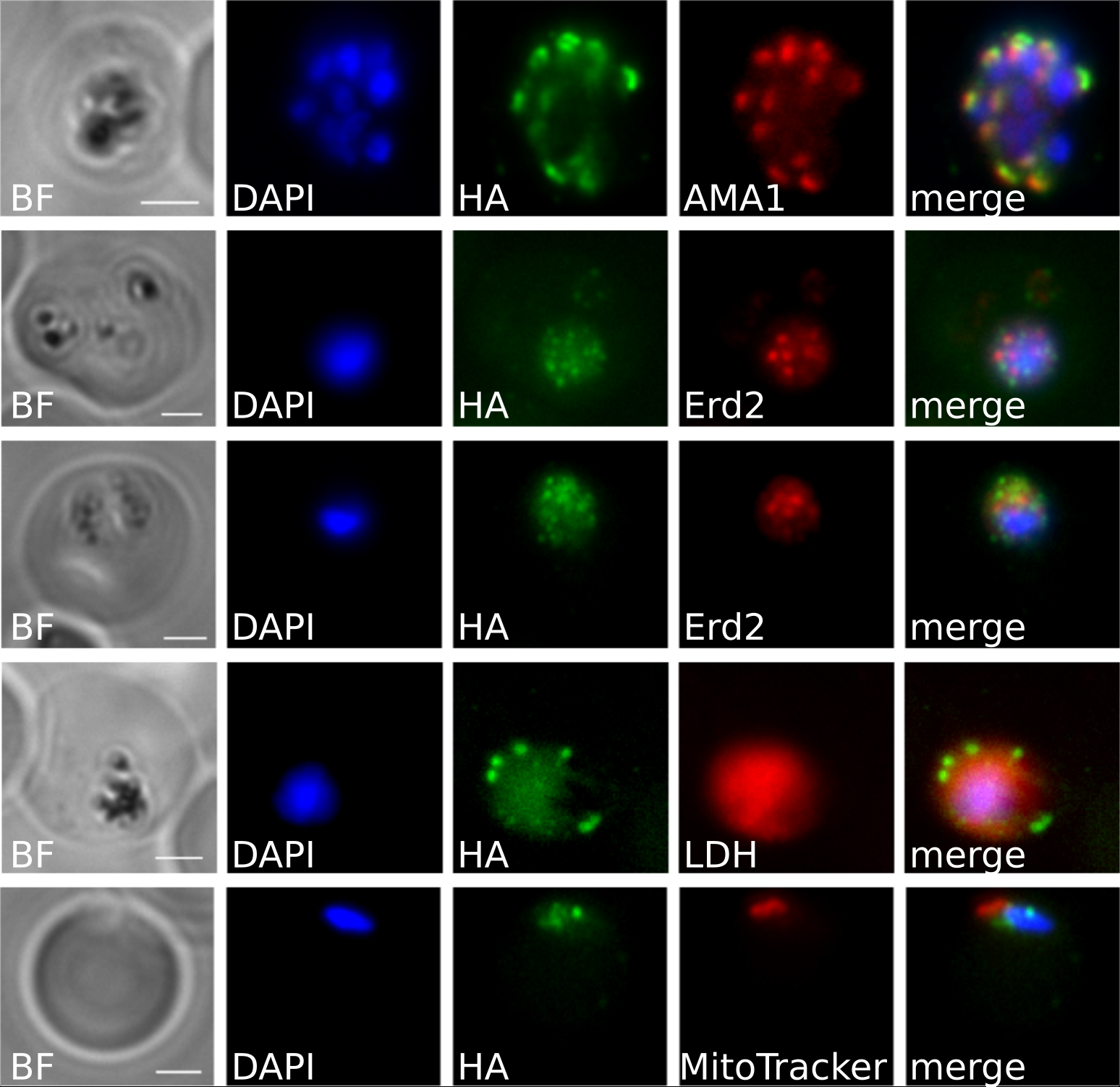

Supplement: S5 Fig — PVX_003539 was localised using either a rat or a rabbit α-HA antibody. Both α-HA antibodies were conjugated with an AlexaFluor® 488 secondary. Costaining markers AMA1, ERD2 and LDH were conjugated with an AlexaFluor® 680 secondary antibody. MitoTracker CMXRos emits at 599 nm. Scale bar 2 μm. (TIF) [file pntd.0007470.s005.tif]

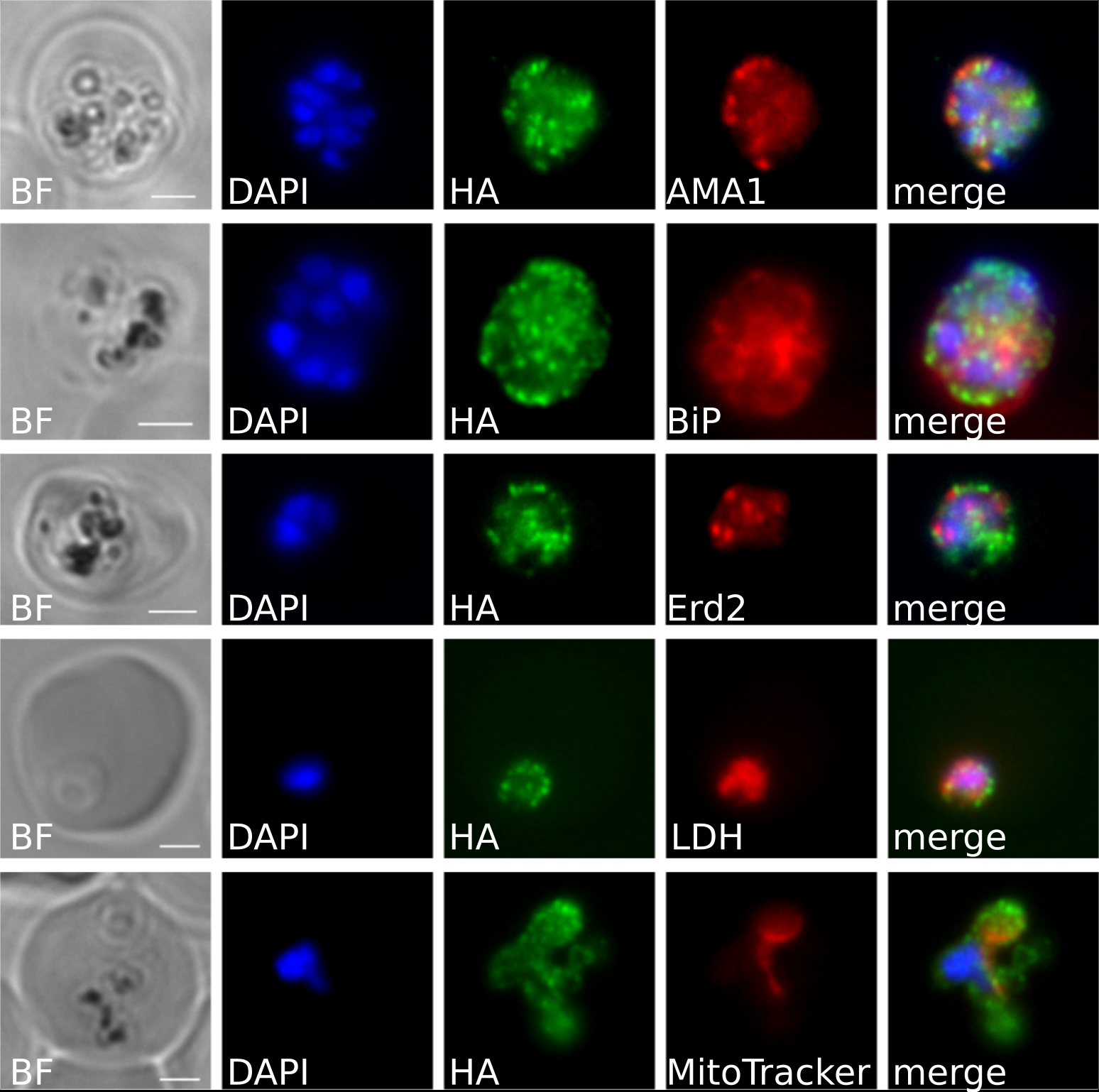

Supplement: S6 Fig — PVX_122995 was localised using either a rat or a rabbit α-HA antibody. Both α-HA antibodies were conjugated with an AlexaFluor® 488 secondary. Costaining markers AMA1, BiP, ERD2 and LDH were conjugated with an AlexaFluor® 680 secondary antibody. MitoTracker CMXRos emits at 599 nm. Scale bar 2 μm. (TIF) [file pntd.0007470.s006.tif]

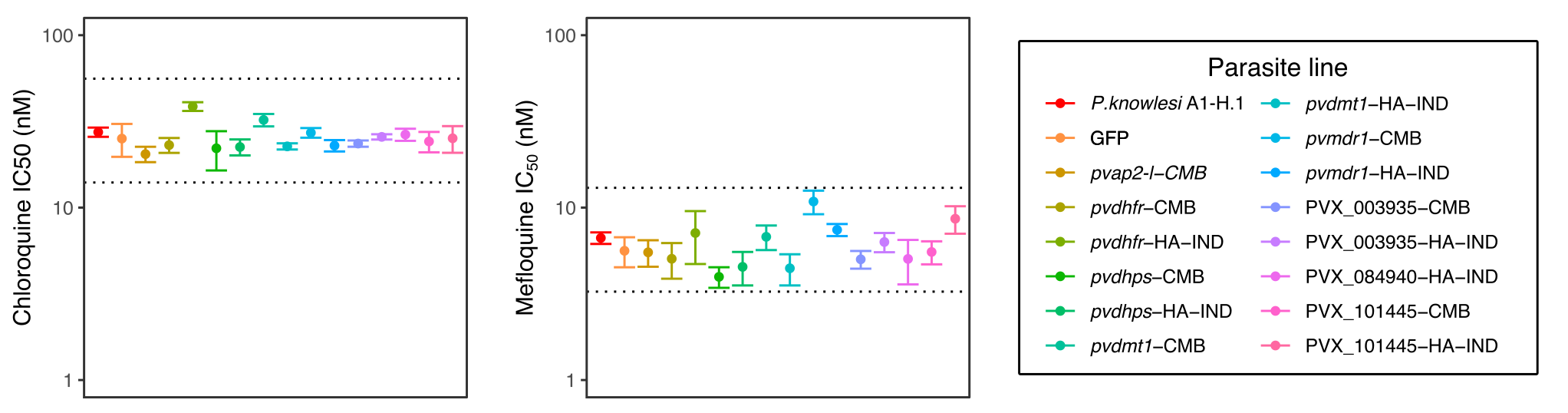

Supplement: S7 Fig — The error-bars represent the 95% confidence interval and a two fold window around the P. knowlesi strain A1-H.1 IC50 is indicated with dotted lines. (TIF) [file pntd.0007470.s007.tif]
